# Different patterns of Australian adults' knowledge of foods and nutrients related to metabolic disease risk

**DOI:** 10.1017/jns.2014.12

**Published:** 2014-08-13

**Authors:** Anthony Worsley, Wei C. Wang, Stephanie Byrne, Heather Yeatman

**Affiliations:** 1 School of Exercise and Nutrition Sciences, Deakin University, Burwood, VIC, Australia; 2 School of Health Sciences, University of Wollongong, Wollongong, NSW, Australia

**Keywords:** Nutrition knowledge, Attitudes toward food, Chronic diseases, Demographics, Latent class analysis, Surveys, Australia, LCA, latent class analysis

## Abstract

A nationwide survey of 2022 consumers was conducted in Australia in late 2011. A short list of questions about knowledge of the nutrient composition of common foods was administered along with questions about the respondents' food attitudes, demographics, school education and dieting practices. Overall, the results showed that nutrition knowledge was relatively high. Latent class analysis showed two groups of consumers with ‘high’ and ‘low’ knowledge of nutrition. Higher knowledge was positively associated with age, female sex, university education, experience of home economics or health education at school, having a chronic disease, and attitudes to food issues, and negatively with type 1 diabetes or the use of diabetes-control diets. The implications of the findings for nutrition communication are discussed.

The prevalence of obesity worldwide has more than doubled over the last three decades^(^^1^^,^^2^^)^. In Australia an estimated 61 % of adults are now overweight or obese^(^^3^^,^^4^^)^. Obesity is a major risk factor for a number of serious chronic diseases including CVD, diabetes and some cancers. These chronic diseases are among the leading causes of death worldwide and are associated with a significant proportion of disability in the world^(^^2^^)^. Consequently, there is a global effort to reduce the prevalence of obesity and prevent the onset and progression of chronic diseases.

Unhealthy dietary patterns, such as high intakes of salt, sugar and fat, and low intakes of fibre, are associated with the development and progression of obesity and many chronic diseases^(^^5^^)^. As energy-dense, nutrient-poor products are increasingly available in the market place^(^^6^^)^, it could be argued that despite the shift towards food-oriented views of nutrition found in recent dietary guidelines^(^^7^^)^ the population's knowledge of the presence of these nutrients in food products is more important today than ever before.

Studies to date have generally shown a weak but significant relationship between nutrition knowledge and dietary behaviours such as fruit, vegetable, fat and fibre intake, and weight loss^(^^6^^,^^8^^–^^15^^)^. Whilst various behavioural models such as the social cognitive theory^(^^16^^)^, Grunert's food-related lifestyle model^(^^17^^)^ and the theory of planned behaviour^(^^18^^)^ indicate that there are many factors involved in food choice, it is likely that nutrition knowledge plays an important part^(^^19^^)^, particularly for those who are motivated to change their dietary patterns.

Identifying population groups that are at risk of having inadequate nutrition knowledge is important to ensure nutrition communication strategies can effectively target these groups. Demographic variation in nutrition knowledge levels has been observed in a number of studies. They show that men and low-socio-economic-status groups are at greater risk of inadequate knowledge than women and high-socio-economic-status groups, respectively^(^^6^^,^^14^^,^^15^^,^^20^^–^^25^^)^. The observed relationship between age and nutrition knowledge has varied between studies. Some have shown a direct association^(^^22^^)^, some have demonstrated an inverse relationship^(^^15^^,^^25^^)^ and others have shown a curvilinear relationship in which middle-aged groups had better knowledge^(^^20^^,^^21^^)^. Other sociodemographic characteristics that have been associated with nutrition knowledge include ethnicity^(^^23^^,^^24^^)^, being on a cholesterol-lowering diet^(^^21^^)^, negatively with smoking status^(^^14^^,^^24^^)^, not being on a special diet^(^^15^^)^, being married or in a *de facto* relationship and number of children^(^^22^^)^.

## Aims

The present study differs from earlier ones in two ways. First, many studies have used a broad range of nutrition knowledge items (for example, Parmenter *et al.*^(^^20^^)^ and Hendrie *et al.*^(^^22^^)^). Our approach is narrower, being confined to analysing knowledge of the presence in selected food products of those nutrients that appear to be related to metabolic disease risk. Second, most studies have assessed levels of nutrition knowledge across populations or demographic subgroups. However, it is likely that within populations experiential and attitudinal factors may influence individuals' knowledge about particular aspects of nutrition. Such characterisation may facilitate better communication with these groups. Therefore the main aim of this cross-sectional study was to identify individuals with differing levels of knowledge of the ‘metabolic nutrient’ composition of some common food products.

## Hypotheses

We expected that age, female sex and higher education would be positively related to food composition nutrient knowledge, as would the presence of children under 18 years (since there would be more occasion to become familiar with nutrition). Again because of their likely greater exposure to nutritional concepts we expected food shoppers to have better knowledge than non-shoppers; those who had undertaken school home economics or health courses or more specialised health or food education courses would have greater knowledge; and that users of ‘special’ diets (for example, vegetarian, diabetic or low-salt diets) would also exhibit greater knowledge. Finally in line with the literature on food involvement^(^^26^^,^^27^^)^ and the theory of planned behaviour^(^^18^^)^, we expected that there would be a positive relationship between attitudes to food issues and nutrition knowledge.

## Materials and methods

### Sampling

The Food Knowledge Survey was an Internet-based survey conducted nationally during November and December 2011. It was designed to determine Australian adults' knowledge of a range of issues related to food including the components of a healthy diet, the nutrient content and health consequences of foods, safe food practices, and a variety of environmental and ethical food issues such as animal welfare and climate change. The survey was conducted by Global Market Insights (GMI), an international market research company. Participants from GMI's database of registered adults living in Australia were invited by email to participate and provided with a link to the survey. Quota sampling was used to ensure that the ages, sex and education and state of residence represented the proportions found in the Australian population. A total of 2022 respondents took part in the survey.

The study was approved by the Deakin University Faculty of Health Human Ethics Committee (HEAG 127-2011).

### Questionnaire

The questionnaire was a combination of newly created questions and modified questions from earlier studies. It covered several areas of food knowledge. The ‘metabolic nutrition’ section of the survey contained sixteen items about the nutrient content of foods. These items were a modified version of a selection of questions from a validated survey developed by Parmenter & Wardle^(^^28^^)^ that have been validated in a sample of Australian adults by Hendrie *et al.*^(^^22^^)^. Respondents were questioned about their knowledge of the saturated fat, dietary fibre, salt and added sugar content of a selection of foods (Table 1).

**Table 1.** Personal background characteristics across latent classes (*n* 2022)

Demographics	% Class 1	% Class 2	% Total
Age (years)			
18–24	10·3	26·1	13·4
25–34	17·8	28·6	19·9
35–44	21·1	24·6	21·8
45–54	23·1	11·9	20·9
55–64	19·5	6·6	17
65 +	8·2	2·3	7·1
Sex			
Male	46·8	65·1	50·4
Education			
Year 11 or less	18·9	22·3	19·6
Completed year 12	17·1	19·5	17·6
Trade and technical qualifications	31·8	28·9	31·3
University	32·1	29·4	31·6
Home economics or health at school			
Yes	58·3	35·4	53·8
Use of diabetes control diets			
Yes	6·8	8·9	7·2
Type 1 diabetes			
Yes	1·5	4·1	2·0
Chronic disease			
Yes	31·5	18·2	28·9

Respondents were also asked about their attitudes to a number of food issues including the nutritional properties of foods, cooking and food preparation, food safety, how to read food labels, how food is grown, processed and distributed, how it is marketed and regulated, food terminology, appropriate serve sizes, environmental impact of food production, fair trade, animal welfare, food security and ethical decision-making. The importance of each of the issues was rated using five-point Likert scales (from 1 = not important to 5 = very important). The responses were summed to form an attitude to food issues score (Cronbach's α = 0·95) which had been developed in an earlier study of experts' views of food information of high relevance to consumers (A-M Parrish, H Yeatman, S Sadegholvad and A Worsley, unpublished results).

In addition, the survey requested background information about the respondents, including their: age, sex, and education status (high school, technical and trade qualifications, university education); the presence of children under 18 years in their household; whether they were the main food shopper or shared the shopping; whether they had attended school home economics or health courses or health or food education courses in years 11 and 12 of secondary school; their consumption of vegetarian, semi-vegetarian or vegan diets; and consumption of low-salt or diabetes-control diets.

### Data analysis

Because of the dichotomous nature of the knowledge items (one option being the correct answer), latent class analysis (LCA) was used to identify different groups of respondents with different levels and types of knowledge. LCA allocates a sample population into mutually exclusive and exhaustive subgroups^(^^29^^)^. In the present study, the response patterns of the sixteen nutrient content knowledge items were subjected to LCA to identify the number of classes (or groups) to which the respondents belonged. LCA was carried out with Mplus version 6·1^(^^30^^)^. The maximum likelihood estimation method was used to adjust the standard errors of the present analyses.

The measurement properties of two and three latent class models were assessed. Several statistical fit indices as well as theoretical considerations were used to do this, including the Akaike information criterion^(^^31^^)^ and the Bayesian information criterion (BIC)^(^^32^^)^. Sample size-adjusted BIC (aBIC)^(^^33^^)^ was also used to determine the number of classes from the competing LCA models^(^^34^^)^. The Vuong-Lo–Mendell–Rubin likelihood ratio test^(^^35^^)^ compares the improvement in fit between neighbouring class models and provides a *P* value that can be used to determine if there is a statistically significant improvement in fit for the inclusion of one more class. Entropy is a measure of classification accuracy^(^^36^^)^, ranging from 0 to 1, higher values indicating better classification. Finally, higher values of the log-likelihood test statistic indicate better model fit.

The present analysis also included predictors of class membership^(^^37^^)^ in which the latent classes were regressed on participants' background characteristics. As noted above, these factors were hypothesised as likely influences on the respondents' item responses. Multinomial logistic regression coefficients for each of the classes were then estimated and compared with the reference class via OR.

## Results

The mean age of the participants (*n* 2022) was 42·6 (sd 14·2) years (Table 2). Of the participants, half were male (50·4 %), and most (59·6 %) were married or living with their partner; two-thirds (66·5 %) did not have children under 18 years living with them; about one-third (31·3 %) had a technical or trade qualification; 31·6 % had a university qualification, and 53·8 % had studied home economics and/or health at school (Table 2). Also, 61·9 % were the primary grocery shopper in their household.

**Table 2.** Probability of latent class membership (%) and item response probabilities (%) within each of the two classes (*n* 2022)

	Class 1	Class 2
Probability of latent class membership	79·7	20·3
Do you think these foods are high or low in added sugar?		
1. Bananas	84·4	52·2
2. Strawberry yoghurt	62·5	25·7
3. Orange juice	86·6	46·3
4. Muesli bar	82·4	31·1
Do you think these foods are high or low in salt (sodium)?		
5. Sausages	96·5	42·2
6. Pasta	58·1	30·1
7. Spinach	94·8	46·3
8. Wholegrain bread	20·6	10·3
Do you think these foods are high or low in dietary fibre?		
9. Cornflakes	65·2	30·1
10. Bananas	75·6	34·6
11. Wholegrain bread	95·3	52·3
12. Fish	67·2	28·9
Do you think these foods are high or low in saturated fat?		
13. Lean red meat	81·8	38·5
14. Whole milk	65·1	29·4
15. Avocado	66·1	35·1
16. Vegetarian pastry	46·1	12·3

**Fig. 1.**
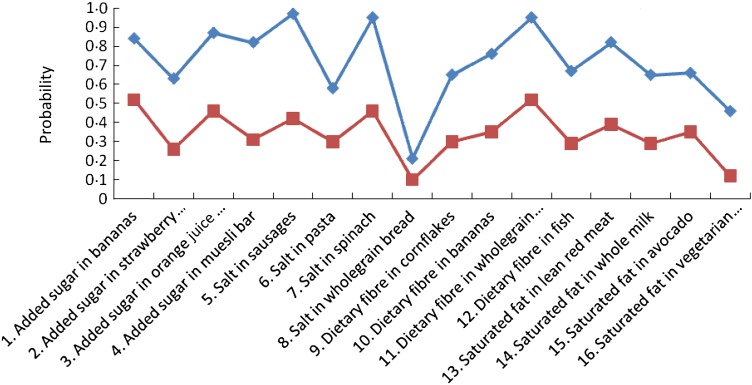
Nutrition knowledge profile of Australian consumers. (–♦–), Class 1; (–■–), class 2.

The participants' nutrition knowledge appeared to be moderately high, with some exceptions. The lowest levels of knowledge pertained to the salt content of wholegrain bread and pasta, the saturated fat content of vegetarian pastry and the added sugar content of strawberry yoghurt (19, 52, 39 and 55 % correctly answering these questions, respectively; see Table 1 and Fig. 1). Knowledge of the nutrient content of some foods was high, for example, the salt content of sausages and spinach and the dietary fibre content of wholegrain bread.

### Latent class results

Inspection of the fit indices and log-likelihood statistics in Table 3 shows that a three-class solution provided the most parsimonious description of respondents' knowledge over the sixteen items. However, the selection of the best-fitting model was subject to not only the statistical fit indices but also the class sizes, theoretical justification, and interpretability. Therefore, a two-class solution was deemed the most appropriate solution for the data.

**Table 3.** Criteria to assess model fit of the latent class analysis models with covariates

Number of classes	Two classes	Three classes	Four classes
Log-likelihood	−17500·418	−17147·676	−15401·922
Number of parameters*	41	66	93
AIC	35082·836	34427·353	30989·844
BIC	35312·922	34797·735	31502·365
aBIC	35182·662	34588·048	31206·907
LMR	0·000	0·000	0·288
Entropy	0·862	0·799	0·793

AIC, Akaike's information criterion; BIC, Bayesian information criterion; aBIC, sample size-adjusted Bayesian information criterion; LMR, Vuong-Lo–Mendell–Rubin likelihood ratio test.

* Number of parameters = K – 1 + K × r + c × (K – 1), where K = number of class, r = number of indicators, and c = number of covariates.

The respondents classified as members of class 1 were more likely to report higher nutrient knowledge than their peers in class 2. In other words, class 1 represents those who performed well on the items (‘good’ knowledge) and class 2 includes those who performed less well (‘poor’ knowledge). Fig. 1 shows the latent class profiles for men and women.

The results of the multinomial logistic regression analyses are presented in Table 4. Class 1 (‘good’ knowledge) is compared with class 2 (‘poor’ knowledge) to interpret the effects of the covariates (listed in Table 4) on the latent class membership. The estimated log odds coefficients and the corresponding log odds CI were then converted into OR and their CI.

**Table 4.** Estimated OR and 95 % CI between the knowledge classes with covariates

	Moderate *v.* low	
Contrast of latent classes	OR	95 % CI
Age	1·65**	1·47, 1·86
Sex	1·59**	1·17, 2·16
Education	1·28**	1·13, 1·45
Home economics or health at school	2·52**	1·87, 3·39
Use of diabetes-control diets	0·50*	0·28, 0·87
Type 1 diabetes	0·34*	0·14, 0·84
Chronic disease	1·67*	1·10, 2·55
Attitudes to food issues	1·71**	1·45, 2·03

*P < 0·05, **P < 0·01 for the multinomial logistic latent class regression weights.

Table 4 shows the OR and their 95 % CI. These results suggest membership of class 1 (‘good’ knowledge) relative to class 2 (‘poor’ knowledge) was associated with: (1) being female (OR 1·59); (2) increased age (OR 1·65); (3) higher education (OR 1·28); (4) having undertaken home economics or health studies at school (OR 2·52); and (5) having a positive attitude to a range of food issues (OR 1·71).

Conversely, individuals who were on a diabetes-control diet (OR 0·50) and those with type 1 diabetes (OR 0·34) were less likely to be members of class 1 (i.e. were more likely to have poor nutrient knowledge) whilst those who had a chronic disease were more likely to have better knowledge (OR 1·67).

Contrary to our hypotheses there was no evidence of any statistically significant relationships between nutrient knowledge and the presence of children under 18 years, marital status; reported hypertension, being a food shopper, having undertaken a specialised course in food or health in years 11 and 12 or at technical college, or following a vegetarian, slimming or other special diet.

## Discussion

The present study showed that respondents' knowledge of ‘metabolic nutrient’ composition of common foods generally was moderately high. The study also showed that there were two groups of consumers: a majority (75 %) with ‘good’ or ‘moderate’ knowledge of these nutrients and another substantial group with lesser knowledge. The latter tended to be less educated, male and younger than the more-knowledgeable group. This is consistent with other studies (for example, Dickson-Spillmann & Siegrist^(^^15^^)^, Parmenter *et al.*^(^^20^^)^ and Hendrie *et al.*^(^^22^^)^). This suggests that the nutrient promotion agenda, though pervasive, has reached only the better-educated, female, older parts of society. This may reflect some disconnection between the mainly declarative metabolic disease agenda (for example, naming harmful nutrients) and the realities of daily life, especially among less-well-off individuals. A promotion agenda that is more relevant to the daily procedures of household food providers (i.e. to food shopping and preparation) might overcome these social disparities. Such a procedural approach involves demonstrating the ways nutritional principles can be used to select foods and prepare meals in specific social contexts.

Novel findings from the present study were that higher levels of knowledge were associated with school home economics and health education, attitudes to food issues and the presence of chronic disease, and the lower levels of knowledge associated with type 1 diabetes and diabetes-control diets.

The greater food composition knowledge of those who had undertaken home economics or health education at school is similar to the findings of McCarthy *et al.*^(^^38^^)^ regarding food safety knowledge. Whilst it is highly likely that more women than men have undertaken home economics and health education at school, it should be noted that the effect observed here was independent of the significant sex effect. If this finding is confirmed in future work it would support the suggestion of Lichtenstein & Ludwig^(^^39^^)^ that home economics or health education during the school years might assist in the prevention of obesity and chronic diseases. The lack of any significant relationships between nutrient knowledge and more specialised food or health education (for example, in years 11 and 12 of high school) may simply reflect the content of these curricula, which tend to emphasise relatively abstract nutritional principles rather than practical selection of foods (for example, Victorian Curriculum and Assessment Authority^(^^40^^)^).

The observed relationships between poorer knowledge and diabetes-control diets and/or having type 1 diabetes are new. These findings require further exploration. They suggest that current dietary protocols may not sufficiently emphasise saturated fats, salt and sugars, related to energy consumption and body weight. This may reflect a focus of diabetic dietary counselling about carbohydrates and glycaemic control, which were not assessed in the present study. Conversely, these findings may indicate that lack of nutrient knowledge could be a risk factor for individuals needing diabetes control. In contrast, the better knowledge of those respondents who suffered from a chronic disease may reflect the Australian medical establishment's pronounced emphasis on the role of fats, fibre and salt in the prevention and amelioration of cerebrovascular disease^(^^41^^)^. Confirmation of these finding is required in future, preferably longitudinal, research.

The positive relationship between the respondents' attitudes towards food issues and knowledge is consistent with both the theory of planned behaviour and the food involvement literature^(^^18^^,^^26^^)^. Attitudes represent the evaluation of beliefs and the more involved individuals are with food (through food purchasing and preparation) the more likely they are to be exposed to information about food, including nutrient information. If consumers are not interested in food issues then it is not likely they will have high levels of nutrient knowledge. Perhaps communication programmes might focus on motivating uninterested consumers to become more interested in food by focusing on issues that do interest them, such as the cost of food, or ways to prepare meals that are consistent with their lifestyles.

The study of nutritional knowledge is important because it may be a necessary factor for population healthy eating, though not wholly sufficient. It is important not to dismiss the importance of nutrition knowledge in the absence of empirical evidence. The significance of the present study is twofold. First, it uses a new technique (LCA) to identify groups of respondents with different levels and types of knowledge – this has not been done previously. Second, it confirms some demographic predictors that were in doubt and identifies others which have not been investigated previously, for example, the likely influence of home economics education.

A related point of significance is that the wider study to which this paper belongs has shown that the various forms of food knowledge (nutrition knowledge, food safety and environmental knowledge) are highly intercorrelated. For example, ‘metabolic nutrition’ knowledge was highly correlated with general nutrition knowledge (*r* 0·92), with general nutrition (*r* 0·90) and with overall food knowledge (*r* 0·87). This suggests that this (or another) short set of items might be used in place of much longer sets of nutrition (and food) knowledge items in surveys and longitudinal studies.

### Implications for nutrition communication and policy

The findings show that there is a substantial minority of the general public who has limited knowledge of the ‘metabolic’ nutrient composition of foods although the majority had relatively high knowledge of the presence of salt, fats, sugars and dietary fibre in foods. However, they can be viewed from quite different perspectives. From the perspective of nutrient-focused health communication the findings suggest that a small group has remained relatively resistant to the mass of nutrient communications over the past 40 years. Under this paradigm more effort might be expended on this ‘stubborn’ group, perhaps through better and more tailored communications. Further, the efforts of agencies such as heart foundations to promote awareness of ‘metabolic nutrients’ during this period can be seen as ‘success’ given the high levels of knowledge of the majority of respondents in the present study.

However, nutrition science is shifting to a more food and dietary focused approach, for example, the Australian Dietary Guidelines are now expressed in terms of foods and food patterns and several researchers have noted the limitation of a nutrient-reductionist approach compared with a food matrix^(^^42^^)^ or food patterns approach^(^^43^^,^^44^^)^. The community's knowledge of ‘metabolic nutrients’ is relatively less important than knowledge of healthy food patterns and the ways required to access and prepare them. From this viewpoint the observed differences in nutrient knowledge seen here are relatively unimportant. Health promotion and communication efforts should focus more on the alteration of daily food practices rather than the provision of nutrient knowledge, depending on life stage. More research is required to examine the influence of ‘food knowledge’ such as health food patterns and food access and transformation skills on food consumption, under the food literacy rubric^(^^45^^)^.

In a similar vein, our findings about the greater metabolic nutrient knowledge of individuals with chronic disease and the lower knowledge of those with diabetes might warrant further exploration of the factors which influenced these respondents' knowledge. Again, in these disease contexts, nutrient knowledge may have quite different practical utility to those encountered in the ‘non-sick’ community. More investigation is required to confirm and extend these findings before any new clinical education recommendations can be proposed.

The findings in this analysis are similar to those from our study of food safety knowledge of the same group of respondents^(^^46^^)^. Two groups were also identified in that study, and age, sex, educational background and school education were associated with different levels of food safety knowledge. However, the findings reported here did not identify any relationships between nutrient knowledge and the use of vegetarian or low-salt diets.

### Limitations and research directions

Whilst no causal relationships can be implied from the present cross-sectional study, the findings do confirm the importance of demographic associations that have been found in many studies. Furthermore, they suggest that both attitudinal and experiential factors may influence nutritional knowledge. Experimental or longitudinal studies are required to examine these findings further in order to establish the relative influence of these factors on both nutrient knowledge and daily food consumption. Another limitation was the small number of foods used in the present study to assess nutrient knowledge. Future studies should examine a broader range of foods. In addition, other facets of nutrition knowledge such as the use of food label information, the nutritional care of infants and children or the dietary care of older individuals could be included in future studies. In particular, better assessment of links between nutrient knowledge, dietary practices and educational experiences is required.

### Conclusions

The public's knowledge of the presence of salt, fats, sugars and dietary fibre in the common foods examined in the present study appears to be quite high. However, about one in four individuals exhibited low levels of nutrient knowledge. The findings suggest that demographic influences, home economics or health education at school, positive attitudes to food issues, and health status are among key factors that may influence this form of nutritional knowledge.
